# ANA IIF Automation: Moving towards Harmonization? Results of a Multicenter Study

**DOI:** 10.1155/2017/6038137

**Published:** 2017-02-21

**Authors:** Stefanie Van den Bremt, Sofie Schouwers, Marjan Van Blerk, Lieve Van Hoovels

**Affiliations:** ^1^Department of Laboratory Medicine, OLV Hospital, Aalst, Belgium; ^2^Department of Laboratory Medicine, GZA Hospitals, Antwerp, Belgium; ^3^Scientific Institute of Public Health (WIV-ISP), Brussels, Belgium

## Abstract

*Background*. Our study aimed to investigate whether the introduction of automated anti-nuclear antibody (ANA) indirect immunofluorescence (IIF) analysis decreases the interlaboratory variability of ANA titer results.* Method*. Three serum samples were sent to 10 laboratories using the QUANTA-Lyser® in combination with the NOVA View®. Each laboratory performed the ANA IIF analysis 10x in 1 run and 1x in 10 different runs and determined the endpoint titer by dilution. One of the three samples had been sent in 2012, before the era of ANA IIF automation, by the Belgian National External Quality Assessment (EQA) Scheme. Harmonization was evaluated in terms of variability in fluorescence intensity (LIU) and ANA IIF titer.* Results*. The evaluation of the intra- and interrun LIU variability revealed a larger variability for 2 laboratories, due to preanalytical and analytical problems. Reanalysis of the EQA sample resulted in a lower titer variability. Diluted endpoint titers were similar to the estimated single well titer and the overall median titer as reported by the EQA in 2012.* Conclusion.* The introduction of automated microscopic analysis allows more harmonized ANA IIF reporting, provided that this totally automated process is controlled by a thorough quality assurance program, covering the total ANA IIF process.

## 1. Introduction

Autoantibodies to nuclear antigens (anti-nuclear antibodies, ANAs) are useful as diagnostic biomarkers for a variety of autoimmune diseases [1–6].

According to the recent recommendations of the American College of Rheumatology, ANA Task Force, the indirect immunofluorescence (IIF) assay on human epidermoid laryngeal carcinoma cells (HEp-2 cells) remains the gold standard for ANA testing [3]. Displaying a multitude of human autoantigens, this substrate enables highly sensitive preidentification of ANAs and the determination of their titers. The rather large variability in ANA titers between laboratories running HEp-2 ANA IIF has been documented in (inter)national proficiency testing programs [7, 8] and is not at all surprising given the intermanufacturer variations in the HEp-2 substrate and the fixation process, differences in conjugate and microscope optics, and, most importantly, the subjective reading of the slides [6].

As an attempt to overcome this lack of standardization, manufacturers have developed several computer-aided diagnosis (CAD) systems, which acquire, analyze, and display digital images of stained IIF slides [9, 10]. Currently available automated ANA IIF image analyzing systems (NOVA View (Inova Diagnostics, San Diego, USA), AKLIDES® (Medipan, Berlin, Germany), Zenit G-Sight (Menarini, Florence, Italy), EUROPattern (Euroimmun, Lübeck, Germany), Image Navigator® (Immuno Concepts, Sacramento, USA), and HELIOS® (Aesku, Wendelsheim, Germany)) have already been reviewed extensively [9, 10]. These systems differ in terms of DNA counterstain, software algorithms for IIF detection and pattern recognition, run-time, types of recognized ANA IIF patterns, and their ability to analyze different substrates. Despite these differences, scientific literature suggests that, because they are all able to overcome some of the drawbacks of manual ANA IIF analysis, these systems may contribute to the harmonization of the HEp-2 IIF analysis [6]. However, until now none of the published studies have investigated the degree of harmonization resulting from ANA IIF automation in actual, routine clinical practice.

Our study aimed to investigate if the use of automated ANA IIF image analyzing systems contributes to the comparability of quantitative results in ANA testing by sending 3 serum samples to 10 clinical laboratories using the NOVA View. Harmonization was evaluated in terms of variability in fluorescence intensity on the one hand and ANA IIF titer variability on the other hand.

## 2. Materials and Methods

### 2.1. Sample Preparation and Distribution

Table 1 lists the 3 samples under study and tabulates the clinical diagnosis, the ANA staining pattern and the presence of antibodies to DNA, and extractable nuclear antigens (ENAs). Samples 1 and 2 were prepared by the clinical laboratories of OLV Hospital Aalst and GZA Hospitals Antwerp. Both samples originated from patients with high titer of ANA and were diluted with ANA negative serum targeting a 1/320 ANA IIF reactivity.

Sample 3 originated from a patient with Sjögren's syndrome. In 2012, before the era of ANA IIF automation, it was sent by the Belgian National External Quality Assessment (EQA) Scheme to all Belgian laboratories performing ANA testing. The overall median titer was 1/1280 [11].

The 3 samples were stored at −20°C, packaged in accordance with postal regulations and distributed by overnight mail. All samples tested negative for hepatitis B surface antigen and antibodies to hepatitis C virus and human immunodeficiency viruses 1 and 2. During the period of the multicenter study, all samples were stored at 2–8°C in the participating laboratories.

Ethical committee approval was obtained in both organizing hospitals (Belgian registration numbers Aalst B126201525864 and Antwerp 150908ACADEM).

### 2.2. ANA IIF Methodology

Ten Belgian clinical laboratories (3 university hospitals, 5 nonuniversity hospitals, and 2 private laboratories), using the automated IIF NOVA View instrument voluntarily, participated in the multicenter study. All used the NOVA Lite HEp-2 ANA kit (Inova Diagnostics, Inc., San Diego, USA), which is mandatory for NOVA View and results in dyeing of the slides with two conjugates: FITC (fluorescein isothiocyanate) and DAPI (4′,6-diamidino-2-phenylindole). Sample dilution (1 : 80) and slide processing were carried out automatically on a QUANTA-Lyser (Inova Diagnostics, San Diego, USA) in all laboratories. An overview of the different types of NOVA View, software version, and QUANTA-Lyser and lot numbers of HEp-2 slides and DAPI-conjugate is available online in “Supplementary Material” at https://doi.org/10.1155/2017/6038137. The presented results have been anonymized. None of the mentioned upgrades (software/model) of NOVA View and QUANTA-Lyser results in methodological changes leading to differences in ANA IIF results.

The NOVA View instrument consists of an automated and fully motorized Olympus 1x81 inverted IIF microscope with 4x, 10x, and 40x objectives and dual band DAPI/FITC filters, a LED light source, and a Kappa DX4 digital camera. The LED UV light source is a CoolLed PreciseExcite with excitation wavelengths of 400 nm (DAPI) and 490 nm (FITC). DAPI fluorescence is used by the NOVA View software for localizing the HEp-2 cells and focusing. Thereafter, the image analysis is performed based on the FITC signal. For each well in a slide, three to five images are acquired with both the DAPI and the FITC filter. Using FITC images, the system measures the average intensity in units named as light intensity units (LIU), discriminating between positive and negative samples. The cut-off set by Inova for ANA IIF positivity is 48 LIU. The NOVA View is able to identify and propose five basic fluorescent ANA patterns (homogeneous, speckled, centromere, nucleolar, and nuclear dots) based on software algorithms. Using pattern-specific dilution curves, the measured LIU can be converted in an estimated endpoint titer (single well titer (SWT)).

### 2.3. Analyses

The participants were asked to perform ANA IIF analysis of each sample:

- 10x in 1 run (to estimate the intrarun variability)
- 1x in 10 different runs (to estimate the interrun variability)
- 1x determination of endpoint titer by dilution

For each of these ANA IIF analyses, the participating laboratories registered the LIU, the SWT, and the pattern, both as it was recognized by the NOVA View and after review (and revision when needed) of the digitalized pictures by an experienced supervisor, resembling a routine workflow.

### 2.4. Statistical Analysis

Data were statistically evaluated with MedCalc® software (Version 14.8.1, Software bvba, Ostend, Belgium). Repeated measures analysis of variances (ANOVA) was used to analyze the difference between groups since the same analysis was performed under different conditions on the same samples. *p* values < 0,05 were considered as significant.

## 3. Results

Positive/negative discrimination by the NOVA View was 100% correct for all analyses (*n* = 93 for all 3 samples). The pattern recognition by the NOVA View was pattern-dependent and 96.7%, 98.9%, and 67.7% correct for samples 1, 2, and 3, respectively. After supervisor's review, this increased to 96.7%, 100%, and 100%, respectively.

**Figure 1:**
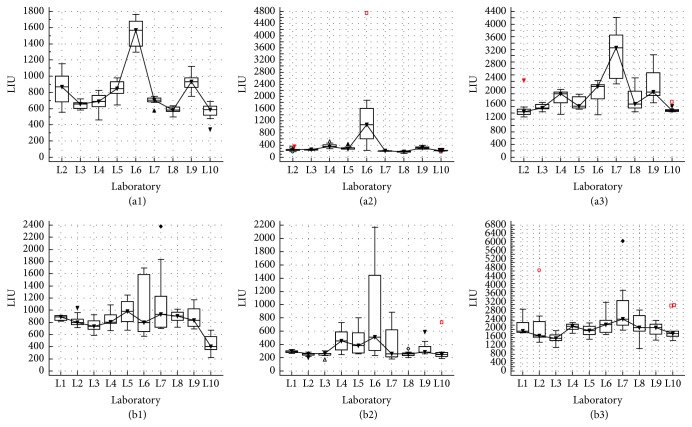
Results of the LIU intrarun variability for samples 1, 2, and 3 ((a1), (a2), and (a3), resp.) and LIU interrun variability for samples 1, 2, and 3 ((b1), (b2), and (b3), resp.) are shown for each individual laboratory before intervention at laboratory 6.

**Figure 2:**
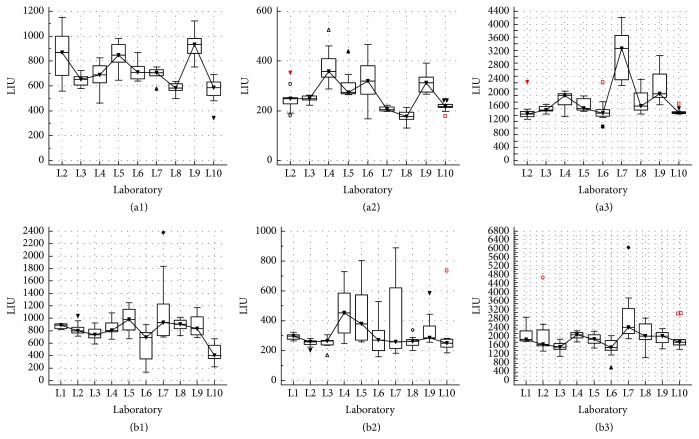
Results of the LIU intrarun variability for samples 1, 2, and 3 ((a1), (a2), and (a3), resp.) and LIU interrun variability for samples 1, 2, and 3 ((b1), (b2), and (b3), resp.) are shown for each individual laboratory after intervention at laboratory 6.

The evaluation of the intra- and interrun LIU variability (Figure 1) revealed a larger variability for laboratory 6 (L6) in comparison with the other participants and a significant (*p* < 0,05) higher median LIU value for samples 1 and 2. For sample 3, the differences were less pronounced. Due to this discordance, the laboratory and the company were immediately informed. An urgent technical intervention showed that the washing module of the preanalytical slide processor was not aligned correctly, resulting in an unequal removal of the conjugate from the wells. After correction, new samples were delivered to L6 and reanalyzed. As shown in Figure 2, the significant difference (*p* < 0,05) of L6 for the median LIU was eliminated by the technical intervention and a good concordance with the other laboratories could be noticed. Also the interrun variability coefficient of L6 improved due to the intervention.

**Figure 3:**
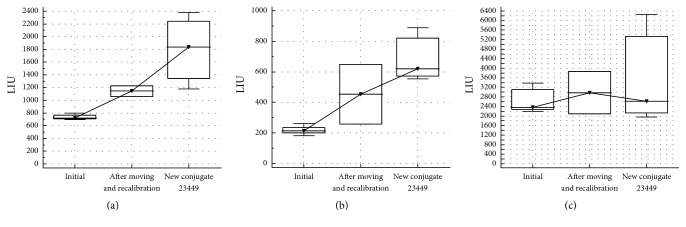
Results of LIU initial interrun variability, interrun variability after relocation and subsequent calibration, and interrun variability after changing to another conjugate lot number for laboratory 7 for samples 1, 2, and 3 ((a), (b), and (c), resp.).

A second laboratory, that is, L7, also showed a higher LIU variability than the other participants, albeit for high LIU values (Figures 1(a3) and 1(b3)). Root cause analysis highlighted 2 major events regarding the ANA IIF analysis in the lab during the survey: a relocation of the NOVA View with a subsequent recalibration, and a change in lot number of conjugate. As shown in Figure 3, both events resulted in remarkable changes in the median LIU (samples 1 and 2) and even in an increase in LIU variability (sample 2 and 3).

**Figure 4:**
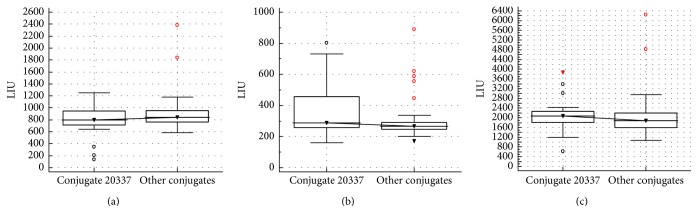
Results of LIU variability for laboratories using conjugate lot number 20337 and laboratories using other conjugate lot numbers for samples 1, 2, and 3 ((a), (b), and (c), resp.).

While the impact of the relocation and recalibration of the NOVA View was specific for L7, the increase in LIU variability caused by the change of conjugate lot number was consistent with a more general observation. All laboratories using the same lot number conjugate, that is, 20337, showed a higher LIU variability than laboratories using the 3 other conjugate lot numbers, but the difference was not significant (Figure 4 for pooled data). The laboratories using this 20337 conjugate lot, that is, L4, L5, L6, L7, and L8, can immediately be identified in Figure 2(b2) (laboratory specific data), characterized by the higher LIU variability. Remarkable is that this variability is less pronounced for samples 1 and 3 (higher LIU). Note that L10 was not included in the conjugate analysis, as they do not register the used conjugate lot number.

**Figure 5:**
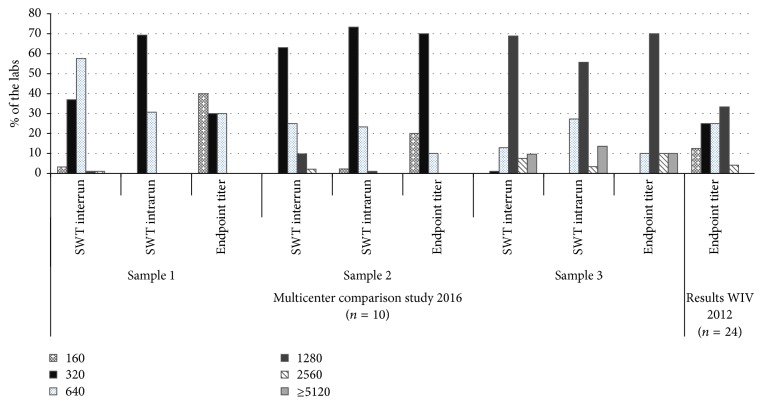
Overview of the reported ANA IIF titers for samples 1, 2, and 3.

The goal of the study was to investigate if automation contributes to the comparability of quantitative results in ANA testing. To this end, sample 3 was included in the study. In 2012, 24 laboratories using the Inova HEp2 substrate performed manual ANA IIF analysis and reported titers ranging within 1 : 160–1 : 2560, with a median ANA IIF titer of 1 : 640, which was comparable with the overall median titer of 1 : 1280 (cfr.  Figure 5). Only 25% of all laboratories reported the median ANA IIF titer of 1 : 640. Analysis of a new aliquot of the original EQA sample in the present multicenter comparison study revealed a lower endpoint titer variability with 70% of the laboratories reporting the median titer of 1 : 1280. In addition, diluted determined endpoint titers on the automated system were similar (+/−1 titer) to the estimated single well titer on the system and the overall median titer of 1 : 1280 as reported by the Belgian EQA in 2012 [11]. The same small variability in endpoint titer reporting was seen for the other samples (Figure 5).

## 4. Discussion

Due to the key position of ANA screening in the serological diagnosis of systemic rheumatic diseases, consistent reproducibility and high quality of HEp-2 cell-based IIF assays are required [4]. However, the visual and therefore subjective evaluation of these assays complicates their standardization. Furthermore, the analysis is labor intensive. Consequently, it does not meet the economic and quality requirements governing current clinical laboratory practice. Contemporary laboratories have to be efficient and highly automated environments. Therefore, in an attempt to overcome the drawbacks of manual ANA IIF analysis, CAD systems were recently developed [12, 13]. Besides the newer CAD system, also the well-established automated slide processing is an important part of ANA IIF analysis.

It is the responsibility of the laboratory to evaluate and control all variables having a potential impact on the “total testing process.” This implicates preanalytical variables like pathology grade variation associated with sample origin, washing or pipetting errors and analytical errors (e.g., reagent lot numbers and calibration errors), but also postanalytical variables (e.g., laboratory information system modification). Our study results reveal a reduction in ANA IIF titer variability between laboratories using the NOVA View but also highlight the importance of quality assurance of the total process, by different problems encountered during the study. Clinically important variability in ANA IIF LIU was shown for L6 due to slide processor washing problems, resulting in extended conjugate incubation times and significant higher LIU values. Calibration error and conjugate lot changes also revealed clinical significant LIU shifts for L7.

The implementation of CAD systems enables the introduction of objective internal control material (iQC) procedures to monitor the total ANA IIF process. Quality assurance can rely on the daily follow-up of LIU values of positive and negative iQC measurements based on the traditionally used Westgard multirules [14, 15]. Regarding those iQC materials, it is important to emphasize that they should assure the whole ANA IIF analysis process, from dilution up to result interpretation, which is not always the case when only company iQC materials are used. Secondly, the effect of major variables (e.g., conjugate changes) is not shown at iQC samples with higher LIU values. Positive iQC samples around the cut-off of positivity reveal the most information. Our study shows already a considerable LIU variability with one type of conjugate. Not only the LIU target value will be affected by a new lot number of conjugate, but also the LIU variation seems to be affected. We anticipate that, besides a paired statistical analysis of the LIU values of a set of routine samples with current and new conjugate lot number, also an intrarun imprecision analysis has to be performed by the autoimmune laboratory, before acceptance in routine ANA IIF practice. Both analyses are of additional value besides the current recommendation of at least 85% numerical equivalent results [15]. However, there is a need for universally accepted guidelines on how to perform lot switch evaluations and consensus criteria for acceptability [16]. Finally, it is worthwhile to include a quality marker for the whole ANA IIF testing process in the daily routine iQC analysis, for example, median LIU for every routine run [17]. This marker is independent on the sample position used and is of added value in the quality management of automated ANA IIF, when initial lot-to-lot comparison protocols fail to detect a change [16].

As the evaluations of neither standardized positive/negative discrimination, neither pattern recognition, were goals of our current study, the samples used were strongly ANA IIF positive with clearly defined and monospecific patterns. The interlaboratory positive/negative discrimination should be a goal for a new study. Enlarging the study scope with other CAD systems on the daily market will even give a more realistic view on the ANA IIF variability burden introduced by the availability of different HEp-2 kits.

Recognizing mixed or “novel” fluorescence patterns remains a challenge for every CAD system, necessitating expert review [9]. This difficulty is even more accentuated by differences in fixative used for different HEp-2 cell preparations, causing a high variability in cell and nuclear morphology which results in significant variability of staining patterns on the ANA HEp-2 IIF substrates obtained with slides from different manufacturers [7, 18]. This challenge has led to a proposed consensus nomenclature for IIF patterns available on the international consensus of anti-nuclear antibody pattern (ICAP) website (http://www.ANApatterns.org). The ultimate goal with the establishment of the ICAP is to promote harmonization of ANA test nomenclature, as well as interpretation guidelines for ANA testing, thereby optimizing usage in patient care [19]. CAD systems offer the opportunity to easily apply this standardized nomenclature in routine autoimmune laboratories.

## 5. Conclusion

Through the introduction of automated microscopic analysis, more harmonized ANA IIF reporting becomes feasible, provided that this totally automated process is controlled by a thorough quality assurance program. This program implies the necessity of validation after recalibration and lot number changing of the most critical reagents (substrate and conjugate) and a continuous internal quality control that covers the total ANA IIF process, from pre- to postanalytical phase. Extending the multicenter comparison to all automated IIF microscopes available nowadays is necessary to address the uniformity of the global ANA IIF analysis.

## Supplementary Material

Overview of the different types of NOVA View, software version, and QUANTA-Lyser and lot numbers of HEp-2 slides and DAPI-conjugate used in the multicenter study.

## Figures and Tables

**Table 1:** Summary of the main sample characteristics of the samples included in the study.

	Sample 1	Sample 2	Sample 3
Disease	Crohn's disease (Remicade therapy)	Unknown	Sjögren's syndrome
Diagnosis/follow-up	Follow-up	Diagnosis	Unknown
Origin	Diluted native OLV patient sample	Diluted native GZA and OLV patient sample	Native patient sample, used by WIV (EQA SN/11641)
Identified autoantibodies^§^	None	CENP-B	SSA/Ro60 + SSB/La
Indirect immunofluorescence pattern on HEp-2	Homogeneous	Centromere	Speckled

Diagnosis/Follow-up = at time of diagnosis versus follow-up of known diagnosis; OLV = Onze Lieve Vrouw Hospital Aalst; GZA = GasthuisZusters Hospital Antwerp; WIV = Scientific Institute of Public Health Brussels; EQA = extern quality control; SN = sample number. ^§^Tested for dsDNA, nRNP/Sm, Sm, SSA/Ro60, Ro52, SSB/La, Scl-70, Jo-1, PCNA, PM-Scl, Rib-P, and CENP-B IgG.
