# Supplementary material for: ANA IIF Automation: Moving towards Harmonization? Results of a Multicenter Study
Source: J Immunol Res. 2017 Feb 21;2017:6038137. doi: 10.1155/2017/6038137 (PMC5339452; doi:10.1155/2017/6038137)
Supplement: Supplementary file 1 — Overview of the different types of NOVA View, software version, and QUANTA-Lyser and lot numbers of HEp-2 slides and DAPI-conjugate used in the multicenter study. [file 6038137.f1.pdf]

Supplementary material: Overview of the different types of NOVA Views® and QUANTA-Lysers® used by the participating laboratories. Also listed are the lot numbers of the NOVALite kit, HEp-2 slides and DAPI-conjugate used in the ring test evaluation.

Laboratory	Type NOVA View®	Preparation slides	Lot number NOVALite kit	Lot number HEp-2 slides	Lot number DAPI-conjugate
**LAB 1**	NOVA View 1 (software v 1.0.4.3)	Automated – Quantalyser -2	022794Z	021404Z	23449
**LAB 2**	NOVA View 2 (software v 2.0.4.3)	Automated – Quantalyser-240	021368Z	018814Z	20642
**LAB 3**	NOVA View 2 (software v 2.0.4.3)	Automated – Quantalyser -2	022173Z	018834Z	20463
**LAB 4**	NOVA View 2 (software v 2.0.4.3)	Automated – Quantalyser -2	023217Z	021534Z	20337
**LAB 5**	NOVA View 2 (software v 2.0.4.3)	Automated – Quantalyser-160	023217Z	021534Z	20337
**LAB 6**	NOVA View 2 (software v 2.0.4.3)	Automated – Quantalyser -2	023217Z<br>028954Z	021534Z<br>027764Z	20337
**LAB 7**	NOVA View 2 (software v 2.0.4.3)	Automated – Quantalyser -2	021368Z	018814Z	20337<br>23449
**LAB 8**	NOVA View 2 (software v 2.0.4.3)	Automated – Quantalyser -2	022794Z	021404Z	20463
**LAB 9**	NOVA View 1 (software v 1.0.4.3)	Automated – Quantalyser-160	NA*	021404Z<br>021534Z	20463<br>20337
**LAB 10**	NOVA View 1 (software v 1.0.4.3)	Automated – Quantalyser -2	021401Z	018826Z	NA*

*NA = not available
